# Supplementary material for: Unveiling post-vaccination proteomic signatures in SARS-CoV-2 infection-naïve individuals associated with Omicron breakthrough infections
Source: PLoS One. 2026 May 11;21(5):e0347602. doi: 10.1371/journal.pone.0347602 (PMC13160346; doi:10.1371/journal.pone.0347602)
Supplement: S1 Fig — (DOCX) [file pone.0347602.s001.docx]

**Unveiling Post-Vaccination Proteomic Signatures in SARS-CoV-2 Infection-Naïve Individuals Associated with Omicron Breakthrough Infections.**

Yiwen Liu PhD^1^ ^†^; Eric Lu BS^2^ ^†^; Katherine D. Ellingson PhD^1^; James Hollister MS^1^; Tuo Liu MS^3^; Wadana Hamzazai MPH^1^; Shawn C. Beitel MSc^3^; Alberto J. Caban-Martinez, DO, PhD, MPH^4^; Manjusha Gaglani, MBBS^5^; Allison L. Naleway, PhD^6^; Lauren E.W. Olsho, PhD^7^; Andrew L. Phillips, MD, MOH^8^; Natasha Schaefer Solle, RN, PhD^9^; Harmony L. Tyner, MD, MPH^10^; Sarang K. Yoon, DO, MOH^8^; Karen Lutrick PhD^11^; Jefferey L. Burgess MD MS MPH^3*^

Author affiliations

1. Department of Epidemiology and Biostatistics, Mel and Enid Zuckerman College of Public Health, University of Arizona, Tucson, AZ, USA

2. Department of Biological Engineering, Massachusetts Institute of Technology, Cambridge, MA, USA

3. Department of Community, Environment and Policy, Mel and Enid Zuckerman College of Public Health, University of Arizona, Tucson, AZ, USA

4. Department of Public Health Sciences, University of Miami, Miller School of Medicine, Miami, FL

5. Baylor Scott & White Health, Temple, Texas and Baylor College of Medicine, Temple Texas.

6. Kaiser Permanente Center for Health Research, Portland, OR

7. Abt Global LLC, Rockville, MD

8. Division of Occupational and Environmental Health, Spencer Eccles Fox School of Medicine, Rocky Mountain Center for Occupational and Environmental Health, Salt Lake City, UT

9. Department of Medicine, University of Miami, Miller School of Medicine, Miami, FL

10. Dartmouth Hitchcock Medical Center, Lebanon, NH

11. Department of Family and Community Medicine, College of Medicine – Tucson, University of Arizona, Tucson, AZ, USA

* Corresponding Author

† These authors contributed equally to this work

**Supporting Information**


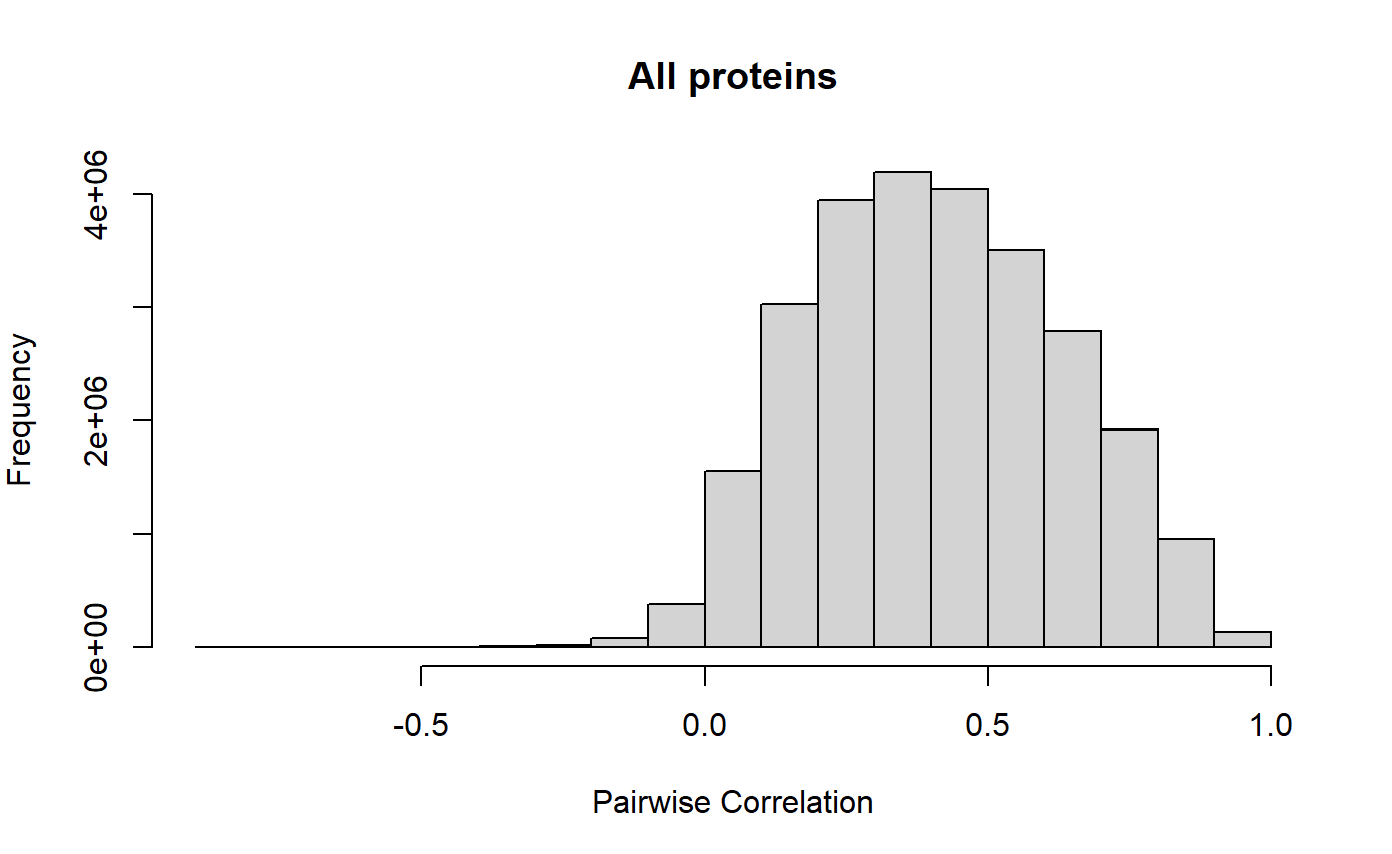


Figure S1. Histogram of pairwise correlations between log2 RFU of proteins showing mostly moderate to high correlation.
