# Supplementary material for: Unveiling post-vaccination proteomic signatures in SARS-CoV-2 infection-naïve individuals associated with Omicron breakthrough infections
Source: PLoS One. 2026 May 11;21(5):e0347602. doi: 10.1371/journal.pone.0347602 (PMC13160346; doi:10.1371/journal.pone.0347602)
Supplement: S1 Table — From left to right, columns correspond to Entrez gene name, regression coefficients, odds ratio, standard error of coefficients, z-score of coefficients, and p-value of coefficients, respectively. (DOCX) [file pone.0347602.s002.docx]

**Unveiling Post-Vaccination Proteomic Signatures in SARS-CoV-2 Infection-Naïve Individuals Associated with Omicron Breakthrough Infections.**

Yiwen Liu PhD^1^ ^†^; Eric Lu BS^2^ ^†^; Katherine D. Ellingson PhD^1^; James Hollister MS^1^; Tuo Liu MS^3^; Wadana Hamzazai MPH^1^; Shawn C. Beitel MSc^3^; Alberto J. Caban-Martinez, DO, PhD, MPH^4^; Manjusha Gaglani, MBBS^5^; Allison L. Naleway, PhD^6^; Lauren E.W. Olsho, PhD^7^; Andrew L. Phillips, MD, MOH^8^; Natasha Schaefer Solle, RN, PhD^9^; Harmony L. Tyner, MD, MPH^10^; Sarang K. Yoon, DO, MOH^8^; Karen Lutrick PhD^11^; Jefferey L. Burgess MD MS MPH^3*^

Author affiliations

1. Department of Epidemiology and Biostatistics, Mel and Enid Zuckerman College of Public Health, University of Arizona, Tucson, AZ, USA

2. Department of Biological Engineering, Massachusetts Institute of Technology, Cambridge, MA, USA

3. Department of Community, Environment and Policy, Mel and Enid Zuckerman College of Public Health, University of Arizona, Tucson, AZ, USA

4. Department of Public Health Sciences, University of Miami, Miller School of Medicine, Miami, FL

5. Baylor Scott & White Health, Temple, Texas and Baylor College of Medicine, Temple Texas.

6. Kaiser Permanente Center for Health Research, Portland, OR

7. Abt Global LLC, Rockville, MD

8. Division of Occupational and Environmental Health, Spencer Eccles Fox School of Medicine, Rocky Mountain Center for Occupational and Environmental Health, Salt Lake City, UT

9. Department of Medicine, University of Miami, Miller School of Medicine, Miami, FL

10. Dartmouth Hitchcock Medical Center, Lebanon, NH

11. Department of Family and Community Medicine, College of Medicine – Tucson, University of Arizona, Tucson, AZ, USA

* Corresponding Author

† These authors contributed equally to this work

**Supporting Information**

Table S1. Unpenalized conditional logistic regression model outputs for HS proteins. From left to right, columns correspond to Entrez gene name, regression coefficients, odds ratio, standard error of coefficients, z-score of coefficients, and p-value of coefficients, respectively.

| **Name** | **coef** | **exp_coef** | **coef_se** | **coef_z** | **coef_p** |
| --- | --- | --- | --- | --- | --- |
| LGALS1 | 1.9078 | 6.7381 | 0.5978 | 3.1912 | 0.0014 |
| PDLIM4 | 1.3782 | 3.9679 | 0.4049 | 3.4043 | 0.0007 |
| VAMP2 | 1.2134 | 3.3651 | 0.3698 | 3.2816 | 0.0010 |
| GALNT10 | 1.2096 | 3.3520 | 0.5886 | 2.0550 | 0.0399 |
| SELE | 0.8431 | 2.3236 | 0.3616 | 2.3318 | 0.0197 |
| PLS1 | 0.7875 | 2.1980 | 0.4793 | 1.6432 | 0.1003 |
| HAVCR2 | 0.7875 | 2.1978 | 0.3470 | 2.2692 | 0.0233 |
| CXCL3 | 0.7510 | 2.1192 | 0.9559 | 0.7857 | 0.4321 |
| HOXA5 | 0.7211 | 2.0568 | 0.7363 | 0.9795 | 0.3274 |
| HAUS1 | 0.6516 | 1.9186 | 0.5852 | 1.1134 | 0.2655 |
| SHKBP1 | 0.6476 | 1.9110 | 0.3237 | 2.0010 | 0.0454 |
| CHRD | 0.6175 | 1.8542 | 0.2547 | 2.4239 | 0.0154 |
| L1CAM | 0.5700 | 1.7682 | 0.3094 | 1.8424 | 0.0654 |
| IL7 | 0.5212 | 1.6840 | 0.2461 | 2.1173 | 0.0342 |
| TMEM132C | 0.4602 | 1.5845 | 0.2571 | 1.7900 | 0.0735 |
| PCDH12 | 0.3850 | 1.4697 | 0.3535 | 1.0891 | 0.2761 |
| DPEP1 | 0.2792 | 1.3221 | 0.3316 | 0.8420 | 0.3998 |
| IMMP2L | 0.1594 | 1.1728 | 0.3256 | 0.4895 | 0.6245 |
| ATP23 | -0.2022 | 0.8169 | 0.3258 | -0.6206 | 0.5349 |
| RAB3IL1 | -0.5549 | 0.5741 | 0.3544 | -1.5659 | 0.1174 |
| PLVAP | -0.5913 | 0.5536 | 0.4448 | -1.3294 | 0.1837 |
| CCL23 | -0.7234 | 0.4851 | 0.3211 | -2.2529 | 0.0243 |
| CCL19 | -0.9813 | 0.3748 | 0.3463 | -2.8336 | 0.0046 |
| THSD7A | -1.0916 | 0.3357 | 0.3152 | -3.4633 | 0.0005 |
| CXCL2 | -1.1877 | 0.3049 | 0.9051 | -1.3122 | 0.1894 |
| NDRG1 | -1.3137 | 0.2688 | 0.5727 | -2.2940 | 0.0218 |
| ZC4H2 | -2.3256 | 0.0977 | 0.6827 | -3.4066 | 0.0007 |
| LCE3B | -2.3661 | 0.0939 | 0.8764 | -2.6997 | 0.0069 |
