# Supplementary material for: Unveiling post-vaccination proteomic signatures in SARS-CoV-2 infection-naïve individuals associated with Omicron breakthrough infections
Source: PLoS One. 2026 May 11;21(5):e0347602. doi: 10.1371/journal.pone.0347602 (PMC13160346; doi:10.1371/journal.pone.0347602)
Supplement: S2 Table — From left to right, columns represent pathway name, p-values computed for the enrichment of each pathway, FDR adjusted p-values, HS proteins contributing to the pathway enrichment, and parent database of each pathway, respectively. P-values for GO-BP and GO-MF database pathways are computed from overrepresentation analysis while KEGG pathways are computed from SPIA (see methods for details). (DOCX) [file pone.0347602.s003.docx]

**Unveiling Post-Vaccination Proteomic Signatures in SARS-CoV-2 Infection-Naïve Individuals Associated with Omicron Breakthrough Infections.**

Yiwen Liu PhD^1^ ^†^; Eric Lu BS^2^ ^†^; Katherine D. Ellingson PhD^1^; James Hollister MS^1^; Tuo Liu MS^3^; Wadana Hamzazai MPH^1^; Shawn C. Beitel MSc^3^; Alberto J. Caban-Martinez, DO, PhD, MPH^4^; Manjusha Gaglani, MBBS^5^; Allison L. Naleway, PhD^6^; Lauren E.W. Olsho, PhD^7^; Andrew L. Phillips, MD, MOH^8^; Natasha Schaefer Solle, RN, PhD^9^; Harmony L. Tyner, MD, MPH^10^; Sarang K. Yoon, DO, MOH^8^; Karen Lutrick PhD^11^; Jefferey L. Burgess MD MS MPH^3*^

Author affiliations

1. Department of Epidemiology and Biostatistics, Mel and Enid Zuckerman College of Public Health, University of Arizona, Tucson, AZ, USA

2. Department of Biological Engineering, Massachusetts Institute of Technology, Cambridge, MA, USA

3. Department of Community, Environment and Policy, Mel and Enid Zuckerman College of Public Health, University of Arizona, Tucson, AZ, USA

4. Department of Public Health Sciences, University of Miami, Miller School of Medicine, Miami, FL

5. Baylor Scott & White Health, Temple, Texas and Baylor College of Medicine, Temple Texas.

6. Kaiser Permanente Center for Health Research, Portland, OR

7. Abt Global LLC, Rockville, MD

8. Division of Occupational and Environmental Health, Spencer Eccles Fox School of Medicine, Rocky Mountain Center for Occupational and Environmental Health, Salt Lake City, UT

9. Department of Medicine, University of Miami, Miller School of Medicine, Miami, FL

10. Dartmouth Hitchcock Medical Center, Lebanon, NH

11. Department of Family and Community Medicine, College of Medicine – Tucson, University of Arizona, Tucson, AZ, USA

* Corresponding Author

† These authors contributed equally to this work

**Supporting Information**

Table S2. Pathway enrichment significance and membership. From left to right, columns represent pathway name, p-values computed for the enrichment of each pathway, FDR adjusted p-values, HS proteins contributing to the pathway enrichment, and parent database of each pathway, respectively. P-values for GO-BP and GO-MF database pathways are computed from overrepresentation analysis while KEGG pathways are computed from SPIA (see methods for details).

| **Pathway** | **p** | **FDR** | **Selected Proteins** | **DB** |
| --- | --- | --- | --- | --- |
| chemokine-mediated signaling pathway | 0.00013 | 0.046122 | CXCL2/CXCL3/CCL23/CCL19 | GO-BP |
| response to chemokine | 0.000148 | 0.046122 | CXCL2/CXCL3/CCL23/CCL19 | GO-BP |
| cellular response to chemokine | 0.000148 | 0.046122 | CXCL2/CXCL3/CCL23/CCL19 | GO-BP |
| neutrophil chemotaxis | 0.000438 | 0.101964 | CXCL2/CXCL3/CCL23/CCL19 | GO-BP |
| neutrophil migration | 0.000819 | 0.118064 | CXCL2/CXCL3/CCL23/CCL19 | GO-BP |
| positive regulation of leukocyte cell-cell adhesion | 0.000922 | 0.118064 | IL7/LGALS1/HAVCR2/SELE/CCL19 | GO-BP |
| granulocyte chemotaxis | 0.001 | 0.118064 | CXCL2/CXCL3/CCL23/CCL19 | GO-BP |
| leukocyte migration | 0.001045 | 0.118064 | CXCL2/CXCL3/SELE/CCL23/CCL19/PLVAP | GO-BP |
| immunological synapse formation | 0.00114 | 0.118064 | HAVCR2/CCL19 | GO-BP |
| response to nitric oxide | 0.001608 | 0.132348 | DPEP1/CCL19 | GO-BP |
| positive regulation of cell-cell adhesion | 0.001727 | 0.132348 | IL7/LGALS1/HAVCR2/SELE/CCL19 | GO-BP |
| granulocyte migration | 0.001764 | 0.132348 | CXCL2/CXCL3/CCL23/CCL19 | GO-BP |
| positive regulation of cell adhesion | 0.001846 | 0.132348 | CHRD/IL7/LGALS1/HAVCR2/SELE/CCL19 | GO-BP |
| chemokine activity | 4.68E-05 | 0.004918 | CXCL2/CXCL3/CCL23/CCL19 | GO-MF |
| chemokine receptor binding | 0.000104 | 0.005453 | CXCL2/CXCL3/CCL23/CCL19 | GO-MF |
| cytokine activity | 0.001725 | 0.058918 | CXCL2/CXCL3/IL7/CCL23/CCL19 | GO-MF |
| cytokine receptor binding | 0.002244 | 0.058918 | CXCL2/CXCL3/IL7/CCL23/CCL19 | GO-MF |
| CXCR chemokine receptor binding | 0.002981 | 0.062608 | CXCL2/CXCL3 | GO-MF |
| G protein-coupled receptor binding | 0.007715 | 0.135012 | CXCL2/CXCL3/CCL23/CCL19 | GO-MF |
| Viral protein interaction with cytokine and cytokine receptor | 5.09E-06 | 0.000137 | CCL23/CCL19/CXCL2/CXCL3 | KEGG |
| Chemokine signaling pathway | 3.65E-05 | 0.000493 | CXCL2/CXCL3/CCL19/CCL23 | KEGG |
| Cytokine-cytokine receptor interaction | 0.000198 | 0.00178 | IL7/CXCL2/CXCL3/CCL19/CCL23 | KEGG |
| NF-kappa B signaling pathway | 0.019921 | 0.134466 | CXCL2/CXCL3/CCL19 | KEGG |
| TNF signaling pathway | 0.0311 | 0.14168 | CXCL2/CXCL3/SELE | KEGG |
| SNARE interactions in vesicular transport | 0.031484 | 0.14168 | VAMP2 | KEGG |
