# Supplementary material for: Unveiling post-vaccination proteomic signatures in SARS-CoV-2 infection-naïve individuals associated with Omicron breakthrough infections
Source: PLoS One. 2026 May 11;21(5):e0347602. doi: 10.1371/journal.pone.0347602 (PMC13160346; doi:10.1371/journal.pone.0347602)
Supplement: Attachment — Submitted filename: Response to Reviewers.docx [file pone.0347602.s006.docx]

**Response to Reviewers**

Dear Editor and Reviewers,

We thank the Editor and the Reviewers for your careful review of our manuscript and for the constructive and insightful comments. We have revised the manuscript accordingly, and we believe these changes have substantially strengthened the clarity, rigor, and presentation of our work. Below, we provide a point-by-point response to each comment. All changes in the revised manuscript are highlighted in track changes.

***Editor Comments***

*The manuscript entitled "Unveiling Post-Vaccination Proteomic Signatures in SARS-CoV-2 Infection-Naïve Individuals Associated with Omicron Breakthrough Infections" has been reviewed and decision for major revision has been made. The authors need to work on the manuscript and incorporate the suggestions made by the reviewers to further enhance the quality of the manuscript.
1. The authors need to provide the acknowledgement section in the manuscript.*

Thank you for pointing this out. We have added the Acknowledgement section in the manuscript.

*2. Please provide the authors' contributions section in the manuscript.*
Thank you for the comment. We have added the Author’s Contribution section in the manuscript.

***Reviewers’ Comments to the Authors***

***Reviewer #1***

*This manuscript presents a timely and technically sound study examining proteomic profiles in vaccinated, infection-naïve individuals who later experienced Omicron breakthrough infections. The authors identify key protein signatures and immune pathways associated with susceptibility to infection. The topic is highly relevant for understanding correlates of protection in the post-vaccination era, and the dataset appears robust. The study is clearly written and well-organized, but several sections would benefit from additional detail, clarification, and contextual discussion to make the findings more interpretable and broadly impactful.

Comments

1. The introduction provides good background on vaccine immunity and breakthrough infections, but it could more explicitly highlight what unique insight proteomic profiling adds beyond antibody and T-cell studies. Clarifying the central hypothesis why proteomics, and what new biology it reveals would make the motivation stronger.*

We have revised the Introduction accordingly to emphasize the unique insights provided by proteomic profiling. Specifically, we added text highlighting the capacity of the large-scale proteomic analyses to capture comprehensive biological signatures. We also clarified the central hypothesis of the study that vaccinated individuals who experienced Omicron breakthrough infections exhibit distinct plasma proteomic signatures indicating differential host responses. All revised text is highlighted in track changes in the manuscript Introduction section.

*2. The paper would benefit from a more detailed description of the cohort age, sex, vaccination type, timing relative to infection, and infection ascertainment method. It’s also important to specify how breakthrough and control subjects were matched and whether potential confounders were adjusted for in the analysis.*

We thank the reviewer for this suggestion. We have revised the Eligibility Criteria and Case Definition subsection to clarify that in-study infections were ascertained using weekly mid-turbinate nasal specimens that were tested for SARS-CoV-2 using reverse transcription-polymerase chain reaction (RT-PCR) at the Marshfield Clinic Laboratory.

We have added Table S3 in the Supplementary Materials, which provides a detailed breakdown of demographic and health characteristics of the eligible 385 cases and 371 controls prior to matching. The final matched cohort generally matches the characteristics of the broader cohort prior to matching (majority female, non-Hispanic/White, and without any chronic conditions).

The details of matching process were provided in the Matching subsection. More specifically,

“Subjects were matched into pairs of case and control individuals in a 1:1 ratio using a greedy matching algorithm based on the following criteria: study site (Tucson, AZ; Phoenix, AZ; Other location, AZ; Temple, TX; Portland, OR; Duluth, MN; Salt Lake City, UT), sex, race/ethnicity (non-Hispanic/White; non-Hispanic/Black; non-Hispanic/Asian; Hispanic; other race), number of chronic conditions (0, 1, 2, or 3+ chronic conditions), age (±3 years), and time between third vaccine dose and blood draw. If no exact match was found, then the case was excluded from the analysis.”

Because these factors were explicitly controlled through matching, they were not included as covariates in the conditional logistic regression models. We further confirmed that immunosuppression was not imbalanced between groups using Fisher’s exact test.

*3. The proteomic platform (e.g., SOMAscan, Olink, LC–MS/MS) should be specified, along with normalization procedures, quality control measures, and correction for batch effects. Likewise, the rationale for using unpenalized conditional logistic regression should be briefly explained, along with how multiple testing was handled across thousands of proteins.*

The SomaScan Platform was used, and sample processing and data preprocessing steps are mentioned in Methods section Line 169 – 193. Additionally, we used a UMAP plot to investigate if batch effects are present and observed that samples cluster by study sites, but these effects have been accounted for through the sample matching.

We used the elastic-net regularized conditional logistic regression for feature selection in the high-dimensional proteomic data, followed by unpenalized conditional logistic regression to obtain the expected difference in subset of proteins consistently selected through stability selection. A total of 28 proteins were highly selected proteins at the $S\geq500$ threshold and were used to fit the unpenalized model. Multiple testing across thousands of proteins was addressed through the penalized feature selection framework rather than marginal hypothesis testing, thus take into account the correlation among proteins and reducing the risk of false discoveries.

*4. The results table lists significant associations (e.g., LGALS1, PDLIM4, VAMP2, HAVCR2, SELE, CCL19), but the text could elaborate on how these proteins fit together functionally. Are they connected within common chemokine or leukocyte signaling pathways, or do they represent independent immune processes? Providing a concise pathway-level summary would help readers understand the biological implications.*

We appreciate the reviewer’s suggestion. In the revised Results section, we have added a concise pathway-level summary paragraph (line 313 – 322 in manuscript) to illustrate how those proteins fit together functionally. Specifically, we emphasized that our selected proteins repeatedly converge on interconnected chemokine signaling, immune regulation, endothelial activation, and adhesion pathways, indicating coordinated immune processes. We also note that those cytokine- and chemokine-related pathways remains robust across alternative stability thresholds.

*5. Pathway analysis is strong and consistent, highlighting chemokine-mediated signaling and cytokine-receptor interactions. Still, the discussion could better connect these findings to known immune mechanisms during Omicron infection such as* *altered leukocyte trafficking, hyperinflammatory responses, or immune evasion. It would also be valuable to mention whether similar proteomic signatures have been observed in earlier SARS-CoV-2 variants.*

We thank the reviewer for this insightful suggestion. We have expanded the Discussion to explicitly connect our findings with the mechanisms of Omicron infection. Specifically, we discussed how the observed downregulation of chemokines may suggest a state of impaired immune readiness, which could facilitate altered leukocyte trafficking, potentially allowing Omicron to bypass early immune barriers. Furthermore, unlike the hyperinflammatory responses in early SARS-CoV-2 infections, our findings suggest that in vaccinated individuals facing Omicron, the characteristics is more of immune evasion and subtle dysregulation rather than hyperinflammation.

*6. The discussion could emphasize how the identified proteomic markers might be used in practice for example, as predictive biomarkers for breakthrough risk, or to guide booster vaccination strategies. Including a short “limitations and future directions” paragraph (e.g., small cohort size, timing of sampling, or generalizability to other variants) would also strengthen the conclusion.*

In the revised Discussion section, we now expand on the potential application of our findings in practice, including that the identified proteomic signatures may inform the breakthrough infection risk, predictive biomarker development, and booster vaccination strategies (line 401 – 411).

We have also added a limitation and future directions paragraph (line 376 – 395) addressing the sample size constraint, issue of single sampling time point, and generalizability of the results.

*7. The abstract should mention at least one or two quantitative findings (e.g., number of significant proteins or key enriched pathways).*

In the abstract, we included the number of proteins identified through stability selection and the major enriched immune pathways. Below is the Results part of the Abstract:

**“Results:** We identified 28 significant proteins out of over 7,000 candidate proteins. Key findings included downregulated chemokines (CXCL2, CXCL3, CCL19, CCL23) and elevated cytokine IL-7 levels in breakthrough cases, with pathway analysis revealing enrichment in chemokine signaling and cytokine-cytokine interaction pathways. Other key proteins, such as LGALS1, HAVCR2, and SELE were upregulated in breakthrough cases.”

*8.* *Add recent references (2023–2024) on post-vaccination systems-level analyses for better context.*

We thank reviewers for the suggestion. We have added several recent references to better contextualize our approach and findings.

**Reviewer #2**

*This study exhibits high scientific rigor, with careful participant matching, robust statistical methodology (EN-CLR with bootstrap stability selection), and thoughtful pathway-level validation. The analytical design successfully addresses the pitfalls of high-dimensional proteomic data and avoids overreliance on single-model feature selection. The identification of chemokine and cytokine alterations linked to breakthrough infections provides biologically coherent insights consistent with current immunological understanding.
However, few limitations merit consideration.
1. The near-homogeneous population (97% non-Hispanic white, 74% female) and inclusion of primarily healthy frontline workers introduce a substantial healthy worker bias and limit generalizability to more diverse or comorbid populations.*

We agree that the demographic composition of our cohort introduces a healthy worker bias and limits the generalizability of our findings to more diverse or medically complex populations. This limitation is now explicitly acknowledged in the revised Discussion line 383 – 390. At the same time, this relatively homogeneous population also reduced variability from comorbid illness and demographic heterogeneity, providing a controlled setting to evaluate proteomic differences associated with breakthrough infection in an infection-naïve, highly vaccinated cohort. For future studies, it would be essential to include more diverse populations, including individuals with higher comorbidity burden or differing demographic characteristics, to improve the robustness and applicability of these findings.

*2. The single post-vaccination sampling time (≈4 weeks after dose three) provides only a snapshot of immune status, precluding conclusions about temporal dynamics of protein expression or causal directionality.*

We agree that measuring proteomic profiles at a single post-vaccination time point provides only a cross-sectional view of immune status and does not allow assessment of temporal dynamics, trajectory of protein responses, or causal directionality. Our objective in this study was therefore not to infer longitudinal immune evolution but to characterize proteomic signatures associated with breakthrough infection within a well-defined time window when immune responses were relatively stable following the third vaccine dose. We have also clarified this point and acknowledged it as a limitation in the revised Discussion line 381 – 395.

*3. Despite using stability selection, the sample-to-variable ratio remains low (~360 participants vs. >7,000 proteins), increasing the risk of overfitting and spurious associations. Independent validation in an external cohort or replication dataset would greatly strengthen confidence.*

We agree that the sample-to-variable ratio in proteomic analyses poses a substantial risk of overfitting and spurious associations, even when stability selection is applied. To mitigate this risk, our analytic framework incorporated several safeguards, including 1) matched design to reduce extraneous variability, 2) elastic-net regularization to shrink unstable estimates, 3) bootstrapped stability selection to prioritize consistently selected proteins across 1,000 resampled datasets, and 4) subsequent refitting of unpenalized conditional logistic regression models only on proteins demonstrating high selection stability. These steps were chosen specifically to reduce the influence of chance-driven features and improve reproducibility of selected proteins. We have also acknowledged the limitation of lacking external validation dataset in the Discussion and emphasize that further work using independent cohorts is essential to confirm these findings.

*4. The mechanistic inference that downregulated chemokines indicate immune exhaustion is speculative without corroborating cellular or longitudinal data. Functional assays (e.g., T-cell activation, cytokine release) are needed to validate proteomic signatures biologically.*

We appreciate the reviewer’s comment. Our intention was to highlight biologically plausible interpretations consistent with observed pathway enrichment and prior literature, rather than assert a definitive mechanistic explanation. In the revised manuscript, we have clarified that our findings represent associations and explicitly noted that validation using functional immune assays (e.g., T-cell activation/exhaustion profiling, cytokine release studies) and longitudinal sampling will be essential to determine whether these proteomic signatures truly reflect immune exhaustion or other regulatory immune states.

*5. Many enriched GO and KEGG terms overlap heavily in chemokine-related functions, raising the possibility that apparent enrichment reflects pathway redundancy rather than distinct biological processes.*

We appreciate the reviewer’s insightful observation. We agree that GO and KEGG share hierarchical and overlapping pathway structures, and therefore redundancy in chemokine-related enrichment is expected. Rather than indicating spurious findings, we interpret this redundancy as evidence of a consistent biological signal converging across independent databases, molecular function categories, and pathway definitions. To help mitigate inflation from redundant pathway definitions, our analysis incorporated FDR adjustment and complementary approaches (GO BP/MF and KEGG SPIA), with significance reproduced across multiple annotation systems. Furthermore, these pathways consistently reflected chemokine signaling, leukocyte migration, cytokine–receptor interaction, and adhesion biology, indicating biologically coherent processes.

*6. Variables such as prior asymptomatic infection, hormonal status, or medication use are not described but could influence circulating protein levels. Their omission may partially confound observed associations.*

We appreciate the reviewer raising this important point. We agree that factors such as prior asymptomatic infection, hormonal status, and medication use would affect proteomic profiles and thus introduce confounding issues. Our study population was drawn from the HEROES-RECOVER cohort, which included intensive longitudinal surveillance, weekly testing irrespective of symptoms, and baseline testing for antibody evidence of previous infection. In combination, these features of our prospective cohort study materially reduce the likelihood of undetected prior infection. However, detailed hormonal or medication history was not uniformly available for all participants, and therefore these variables could not be incorporated into the current analysis. We have now acknowledged this limitation in the revised manuscript (line 388 – 395). For future studies, more comprehensive clinical characteristics would be included to further delineate these potential influences.

*7. The manuscript would benefit from explicit mention of cross-validation parameters, stability thresholds (why S > 500 was chosen), and sensitivity analyses for model robustness.*

For each bootstrap dataset, the regularization parameter $\lambda$ was determined using 10-fold cross-validation with model deviance as the selection criterion. As a result, the optimal $\lambda$ was data driven and could vary across bootstrap iterations. We have also added justification for our stability threshold. We take the “majority vote” criterion, such that protein selection frequencies were computed across the 1000 bootstrap datasets, and proteins that were selected in majority of the bootstrap models ($S>500$) were considered. This “majority vote” approach is consistent with conventional stability selection principles, prioritizes proteins that demonstrate high reproducibility across resampled datasets and reduced the likelihood of chance-driven inclusion.

In addition, we conducted sensitivity analysis using alternative stability thresholds ($S>400, S>600$) and found that the selected proteins consistently demonstrate an enrichment of chemokine-related pathways. The following sentence (lines 318 - 320) has been added to include to mention this point: “Moreover, cytokine/chemokine-related pathways remain enriched over a range of threshold selection frequencies (S>400 and S>600) used to define the HS protein set.”

*Overall, while the work is methodologically innovative and timely, its translational relevance would be greatly enhanced by validation in more heterogeneous populations, longitudinal sampling, and orthogonal functional assays to confirm causality and biomarker potential. These areas require clarification and improvement before it can be accepted for publication.*
